# Neurobiological Aspects of Face Recognition: The Role of Oxytocin

**DOI:** 10.3389/fnbeh.2018.00195

**Published:** 2018-08-28

**Authors:** Olga L. Lopatina, Yulia K. Komleva, Yana V. Gorina, Haruhiro Higashida, Alla B. Salmina

**Affiliations:** ^1^Department of Biochemistry, Medical, Pharmaceutical, and Toxicological Chemistry, Krasnoyarsk State Medical University named after Prof. V.F. Voino-Yasenetsky, Krasnoyarsk, Russia; ^2^Research Institute of Molecular Medicine and Pathobiochemistry, Krasnoyarsk State Medical University named after Prof. V.F. Voino-Yasenetsky, Krasnoyarsk, Russia; ^3^Department of Basic Research on Social Recognition and Memory, Research Center for Child Mental Development, Kanazawa University, Kanazawa, Japan

**Keywords:** face recognition, oxytocin, social stimuli, autism spectrum disorders, neurotechnologies

## Abstract

Face recognition is an important index in the formation of social cognition and neurodevelopment in humans. Changes in face perception and memory are connected with altered sociability, which is a symptom of numerous brain conditions including autism spectrum disorder (ASD). Various brain regions and neuropeptides are implicated in face processing. The neuropeptide oxytocin (OT) plays an important role in various social behaviors, including face and emotion recognition. Nasal OT administration is a promising new therapy that can address social cognition deficits in individuals with ASD. New instrumental neurotechnologies enable the assessment of brain region activation during specific social tasks and therapies, and can characterize the involvement of genes and peptides in impaired neurodevelopment. The present review sought to discuss some of the mechanisms of the face distinguishing process, the ability of OT to modulate social cognition, as well as new perspectives and technologies for research and rehabilitation of face recognition.

## Introduction

Face perception and recognition are essential elements of social interaction, and represent critical skills acquired early in human life. Socially meaningful information regarding levels of familiarity, attractiveness, and emotional status can be derived from facial recognition, which then shapes behavioral patterns. Loss of the ability to recognize faces is usually associated with impaired neurobiological mechanisms related to visual face perception and/or memory problems. Indeed, alterations in face perception can lead to prominent changes in sociability observed in individuals with severe brain conditions, including autism spectrum disorder (ASD), Turner syndrome, Alzheimer’s disease, depression, and schizophrenia. Particularly, individuals with ASD may exhibit abnormal memory for facial identity, gaze processing, and recognition of emotional facial expressions (Golarai et al., 2006). Although these disturbances can be difficult to characterize, parents of autistic children are able to recognize subtle deficits in face processing that occur within the context of broad autism phenotypes (Yucel et al., 2015). As a result of the neurodegeneration that accompanies Alzheimer’s disease, difficulties in memory retrieval and mental rotation of faces can affect the ability of patients to recognize faces (Adduri and Marotta, 2009). Further, in individuals with depression, the perception of negative faces can be stronger than that for positive faces (Dai and Feng, 2012).

Comprehensive studies have found that neuropeptides correspond to face processing. For instance, the neuropeptide oxytocin (OT) plays a significant role in different types of social behaviors, including face and emotion recognition (Guastella et al., 2008; Bartz et al., 2011). Recently, nasal OT administration has shown therapeutic promise in addressing deficits in social cognition that occur in individuals with psychiatric disorders such as ASD. Some psychological mechanisms underlying face recognition processes have been addressed in previous publications, and thus will not considered here. This review will focus on the ability of OT to modulate social cognition and to ameliorate social impairment that occurs in various disorders, with a focus on ASD.

## Brain Regions Involved in Face Recognition

The detection and recognition of faces have been found to be distinct processes involving neural systems that are not likely implicated in non-social object recognition (Tsao and Livingstone, 2008). Studies conducted from the 1970s to the 1990s revealed that face processing is linked to different brain circuits that are involved in face discrimination, familiar face recognition, and unfamiliar face recognition (Elgar and Campbell, 2001). More recently, studies using Positron-emission tomography (PET) and functional magnetic resonance imaging (fMRI) have attributed the neurobiological basis of face perception impairment to alterations in clusters of face-selective neurons located in the temporal lobe (de Souza et al., 2008) or the fusiform face area, which is part of the fusiform (occipitotemporal) gyrus (Tsao and Livingstone, 2008). Visual face recognition, which requires either simultaneous feature integration or subsequent feature integration, was found to activate the fusiform gyrus without lateralization, such that both hemispheres are equally involved in the spatial and temporal integration of features (James et al., 2010). However, subsequent recent clinical and neuroimaging studies have found that activation of the fusiform face area in the right hemisphere is associated with holistic processing, while activation of the same area in the left hemisphere is associated with analytic processing (de Moraes et al., 2014). This suggests that the advantages for face analysis in the right hemisphere would be lost in cases where an individual is required to perform recognition of inverted faces. Very recently, a right hemispheric dominance in face recognition in humans was confirmed, and the face-selective response was found to be largest in the right middle fusiform gyrus (Jonas et al., 2016).

Almost two decades ago, neurons in the prefrontal cortex were found to selectively respond to faces (Scalaidhe et al., 1999; Nelson, 2001). These face-selective responses were paired with strong interactions and emotional responses within the temporal lobe, hippocampus, and amygdala, which enable encoding, storage, and retrieval of both short-term and long-term memories (Simons and Spiers, 2003). Neurons in the inferior temporal cortex are driven by the contrast and geometrical features of objects. In accordance with the encoding specialization of these neurons, face-selective responses are generated through the modulation of firing rate within a wide spectrum. Thus, multiple different high contrast features can be identified in the target object (Ohayon et al., 2012). Further, single neurons have been found to selectively respond to specific individuals (Quiroga et al., 2005). Experiments with non-human primates have confirmed that face-selective neurons may preserve specific activity patterns induced by face recognition (McMahon et al., 2014). Interestingly, this has been found to correspond to up to 1 year of familiarization.

Within the medial temporal lobe, hippocampal neurons contribute to the recollection of the stimulus, whereas the perirhinal cortex is involved in familiarity-based recognition (Eichenbaum et al., 2007). The occipital face area takes part in detailed face recognition, but is not involved when the fusiform face area is activated by simple discrimination of faces from non-social stimuli (Atkinson and Adolphs, 2011).

**FIGURE 1:**
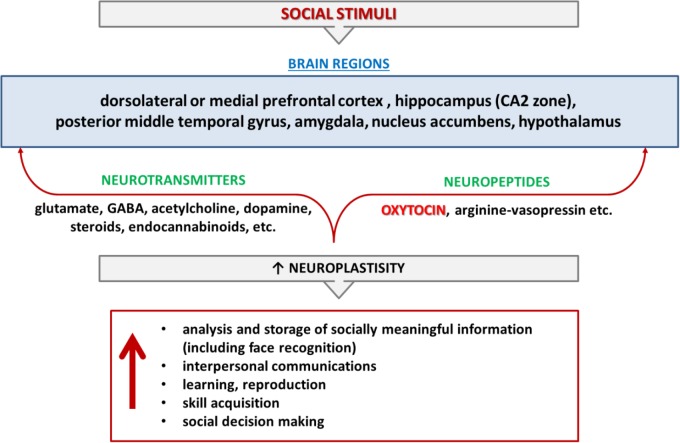
Cognition and memory of social interactions. Cognition and memory of social interactions are controlled by different brain regions and mediated by various neurotransmitters (glutamate, GABA, acetylcholine, dopamine, steroids, endocannabinoids, etc.) and neuropeptides (oxytocin, arginine-vasopressin) in mammalians. Red color indicates the items that are in the review’s focus.

Face-selective neurons have been found in the amygdala, indicating that this region plays an important role in face recognition (Kosaka et al., 2003). Todorov (2012) proposed that the role of the amygdala in face perception is to motivate the brain to pay attention to novel socially meaningful stimuli (faces). This may explain why face recognition is often impaired in patients with amygdala atrophy. Specifically, fMRI studies have revealed that patients with frontotemporal degeneration associated with the affected amygdala do not display appropriate activation for fearful compared with neutral faces (De Winter et al., 2016). Face-specific responses in the amygdala vary depending on the individual, although atypical positive and negative faces are the strongest inducers of amygdala-mediated face perception (Todorov et al., 2013). Several studies have ruled out the possibility that fearful face perception might be linked to anxiety level, as no direct correlation was found between anxiety and threatening face detection (Doty et al., 2014). In individuals with generalized social anxiety disorder, OT simultaneously dampens amygdala reactivity and enhances amygdala functional connectivity with the insula and middle cingulate/dorsal anterior cingulate gyrus during the processing of fearful faces (Gorka et al., 2015). However, in the general population, there is evident variability in the ability to detect fearful faces. Individuals who are able to recognize fearful faces demonstrate more prominent amygdala activity compared with those who are unable to discriminate fearful faces (Pessoa et al., 2006) (**Figure 1**).

Ventral occipitotemporal face-preferential regions are activated regardless of long-term face familiarity, whereas the medial temporal lobe (including the hippocampus and amygdala) and anterior inferior temporal cortex only respond when information regarding familiar faces has been accumulated (Ramon et al., 2015). In children, face-processing tasks do not induce network modulation with the same level of intensity as that observed in adults, indicating that the relevant functional connections do not mature until about 12 years of age (Behrmann et al., 2016).

Translating human social recognition deficits into rodent and non-human primate behavior well-demonstrated sensitive brain regions that responsible for the ability to recognize and distinguish between conspecifics: amygdala (Qi et al., 2018), nucleus accumbens (Mul et al., 2018), hippocampus (Raam et al., 2017; Lin et al., 2018), hypothalamus (Remedios et al., 2017). Cortical and subcortical circuits support efficient social recognition (Rogers et al., 2018).

## Brain Activity and Face Recognition in Humans

Electrophysiological and magnetoencephalographic (MEG) analyses have indicated that face processing might be monitored via several types of synchronized electrical activity in the brain. Specifically, theta (4–8 Hz) and gamma oscillations (28–48 Hz) appear to be helpful for distinguishing the recognition of known (familiar) from unknown faces (Başar et al., 2007). Face perception induces gamma oscillations with a frequency higher than 30 Hz, reflecting the balance of GABA/glutamate-controlled inhibition/excitation in the brain. Such oscillations appear to be larger when a face is presented in comparison with other objects, and thus might be associated with the integration of visual inputs (Engell and McCarthy, 2011; Gao et al., 2013). In general, compared with object recognition, face recognition appears to activate more synchronized neuronal assemblies (Zion-Golumbic et al., 2008).

In young children (up to 8 years old) with ASD, gamma oscillations recorded via EEG and MEG revealed signs of desynchronization (Kikuchi et al., 2013a). In terms of impaired brain functional connectivity in this population, the degree of developmental delay was found to correspond with the degree of excess gamma band oscillations in the frontal area, suggesting aberrant visual perception and cognition. Preserved visual reasoning ability was coupled with rightward lateralization in the functionally connected parietal and temporal regions in children with ASD (5–7 years old) (Kikuchi et al., 2013b). Face perception-induced activation can also be monitored via event-related potential (ERP) components, particularly, the N170, which serves as a marker of face processing in the brain (Feuerriegel et al., 2015).

Brain regions involved in face perception and recognition in normal individuals might not be fully activated in those with ASD who have morphological abnormalities in the amygdala (Pierce et al., 2001). A recent meta-analysis of neuroimaging data indicated that activation patterns in brain regions responsible for social and/or face cognition in ASD patients differ from those in typically developing individuals (Patriquin et al., 2016).

## Oxytocin and Control of Face Recognition in (Patho)Physiological Conditions

Central release of OT is a major regulator of numerous social behavioral processes, including social communication, social recognition, social memory, interpersonal cooperation, and decision-making (Jurek and Neumann, 2018).

Rodent studies well-established that release of OT is controlled by CD38. Being actually NAD^+^-glycohydrolase, CD38 synthesizes cyclic ADP-ribose which is a Ca^2+^-mobilizing second messenger present in OT-producing neurons (Jin et al., 2007). Therefore, researchers have postulated that impaired CD38-mediated OT release might be caused by intracellualar NAD^+^ depletion (i.e., under conditions of oxidative stress or DNA damage and hyperactivation of NAD^+^-consuming DNA repair enzymes), and diminished CD38 expression or lower enzymatic activity may lead to prominent alterations in various aspects of mammalian social behavior (Jin et al., 2007; Higashida et al., 2012; Lopatina et al., 2012). Low CD38 expression is associated with a risk for ASD through impaired OT secretion (Lerer et al., 2010). Expression of CD38 [and single nucleotide polymorphisms (SNPs) in the CD157 gene as a paralog of CD38] is correlated with scores on the Autism Quotient (Chong et al., 2017). Moreover, CD38 SNP (rs3796863) (AA genotype) is associated with elevated plasma OT concentrations and increased levels of suicidal intentions, this is thought to be due to an increased sensitivity to disturbed social relations (McQuaid et al., 2016).

Previous mammalian studies have shown that OT may rescue social behaviors affected by alterations in OT-controlled signaling systems. Long-term social recognition memory in rats involves protein synthesis and OT-dependent long-term depression in the medial amygdala (Gur et al., 2014). Male rats recognized a previously encountered female adult stimulus with a region-dependent contribution of endogenous OT (Lukas et al., 2013). CD38^-/-^ mice demonstrate abnormal social memory and OT administration rescues it (Jin et al., 2007). And impaired social preference in oxytocin deficient mice may be due to severe deficits in social recognition (Tsuda et al., 2018).

Genetic, optogenetic, and pharmacological manipulations proof that oxytocin receptors (OXTRs) signaling is crucial for entrainment of odor to social cues. OT directly impacts the piriform, the olfactory sensory cortex, to mediate social learning and plays a role in both appetitive and aversive social learning (Choe et al., 2015). Retrograde neuronal tracing combined with immunocytochemistry revealed that the OT neurons in the paraventricular nucleus project directly to the CA3 region of the hippocampus (Lin et al., 2017) and optogenetic terminal-specific attenuation confirmed a critical role for aCA2/CA3 outputs to posterior CA1 region of hippocampus for discrimination of social stimuli (Raam et al., 2017).

Dogs are social animals and have been verified to show numerous human-analog social behaviors and essential to OT system research. Intranasal OT enhances dogs’ ability to use human pointing cues in an object choice task (Oliva et al., 2015; Macchitella et al., 2017). And these effects of exogenous OT are breed-specific. For example, Border Collies are looked more at the owner and shifted their gaze more between the sound source and the owner in a potentially dangerous situation and looked longer at the experimenter’s eyes in the ‘Tolerance of prolonged eye contact’ test after OT administration (Kovács et al., 2016).

Nowadays eye tracking method to dogs is viable. Dogs show a greater visual preference for emotionally meaningful face areas (e.g., the eyes as opposed to the neck and the forehead) and a single dose of intranasal OT decreases dogs’ looking to the human faces expressing angry emotional expression (Kis et al., 2017).

Accumulating evidence from non-human primates studies indicates that functional benefit in the neural face recognition system is linked to regulation of OT release and OT action at target cells. Direct administration of OT into amygdala boosts social attention and promotes pro-social decisions in rhesus macaques (Chang et al., 2015). Intranasal treatment (inhaling aerosolized) with OT relaxed social interactions between monkeys and injection of OT into the anterior cingulate gyrus reproduced all of these effects (Jiang and Platt, 2018). Recently, Madrid et al. (2017) conducted an elegant study that found that face recognition for novel faces in young monkeys (3–4 months old) predicted their cerebrospinal fluid OT levels during the subsequent 5 years. A greater preference for novel faces was correlated with higher central OT concentrations, but not blood OT concentrations. The authors suggested that central OT biology is related to individual face perceptual abilities that are necessary for group living. Because this preference was unchanged for 5 years, they also suggested that this trait is stable, which may enable animals to live together over long periods of time.

Human study reveals the administration of OT facilitates face recognition via an increase in the salience of socially important stimuli (Bate et al., 2015). Specifically, well-known human faces are recognized more accurately after OT administration. This appears to be due to an increase in the familiarity of the faces kept in one’s memory. Further, OT improves the recognition of faces with positive emotions. This is likely due to enhanced memory, as opposed to enhanced perception. Almost all of these effects appear to be gender-specific, although OT-induced memory impairment has been documented in both women and men (Herzmann et al., 2013). OT increases the ability of Korean males to recognize positive emotion, and this effect is dose-dependent (Shin et al., 2018). A recent study reported that in women, OT increased the accuracy of recognition of faces displaying angry and happy emotions and reduced the response time to negative emotional faces, while the same dose of OT had no effect in men. This indicates that OT may increase the efficiency of working memory involved in face processing in women (Yue et al., 2018).

**FIGURE 2:**
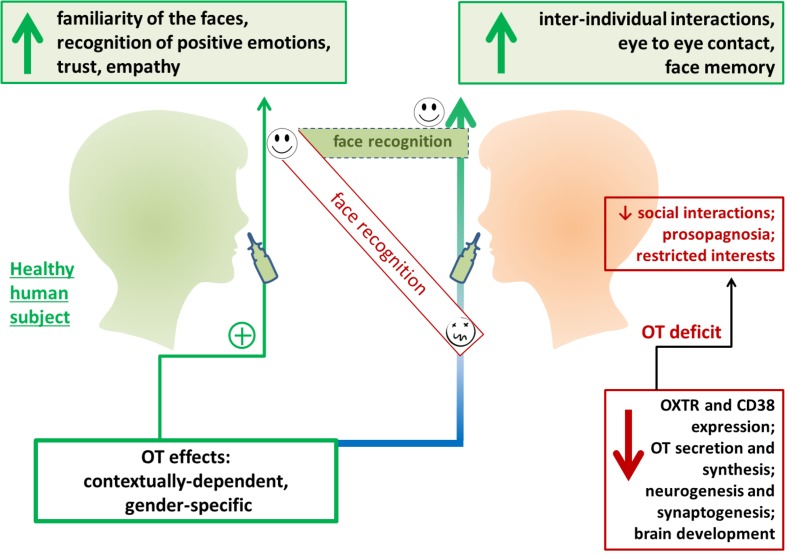
Exogenous oxytocin (OT) facilities face recognition in humans. Intranasal OT administration has been found to improve perception of facial expressions and affect trust in human participants. OT administration has been found to improve inter-individual interactions and communication in individuals with social behavior deficits. OXTR, oxytocin receptor.

Exogenous OT greatly affects face recognition in humans. For instance, administration of OT 40 min before face memory encoding resulted in an improved ability to recognize a face viewed on the previous day, while OT administration had no effect on non-social stimuli (Rimmele et al., 2009) (**Figure 2**). Interestingly, intranasal OT administration in men suppressed the response of the right amygdala to faces with various emotional expressions, indicating that attenuation of amygdala activity could facilitate social behavior, regardless of whether the social stimuli are positive or negative (Domes et al., 2007). However, the opposite effect was observed in women: the same dose of exogenous OT resulted in enhanced amygdala activity in response to fearful faces. This effect was particularly strong in the luteal phase of the menstrual cycle (Domes et al., 2010). Conversely, some data support the absence of any clear association between gender and OT action in terms of face processing. This is presumably because gender-specific differences in face recognition are based on OT-mediated events, thus, women usually recognize all human faces as more positive, but with a significant preference for children’s faces (Proverbio, 2017) (**Figure 2**). Recent study confirmed that females show more accurate facial emotion recognition compared to males and are faster in correctly recognizing facial emotions, but males and females do not differ in the recognition of neutral faces (Wingenbach et al., 2018).

Asperger’s syndrome, which is now classified as a type of ASD, is linked with OT insufficiency (Di Napoli et al., 2014; Domes et al., 2014). This disorder is characterized by alterations in non-conscious and conscious facial emotion recognition that have been measured using current experimental protocols (Chien et al., 2016). The amygdala may be involved in mediating the stimulating effect of exogenous OT in patients with Asperger’s syndrome (Domes et al., 2014).

Deficits in OT secretion are believed to result in autism-like behavioral traits (Neumann, 2008; Higashida et al., 2011). Thus, it is not surprising that a common SNP in the OXTR (rs237887) was found to be associated with the parameters of face recognition memory in 198 European families with at least one child with an ASD diagnosis (Skuse et al., 2014). However, this study used the Warrington recognition memory test for faces, and these findings were not replicated in an additional study with 370 participants that used alternative protocols (Verhallen et al., 2017), specifically, the Cambridge face memory test, Mooney face test, Glasgow face matching test, and the Composite face test. Another SNP in the OXTR (rs7632287) was found to be associated with the ability to recognize faces. Individuals who carry the GA genotype exhibit enhanced amygdala activity during face encoding, as well as enhanced memory (Westberg et al., 2016). Even in typically developing children, SNPs in the OXTR are associated with the parameters of face recognition. A specific combination of SNPs (rs2254298 and rs53576) has been proposed to most efficiently modulate social behaviors (Slane et al., 2014). Particularly, rs2254298 contributes to amygdala volume and the response to salient social cues in face recognition tests (Marusak et al., 2015). Other SNPs in the OXTR dictate the peculiarities of pro-social cooperative behaviors and the functional activity of the amygdala during the emotional face-matching task (Haas et al., 2013; Jurek and Neumann, 2018).

Behavioral tests and fMRI studies have indicated that individuals with ASD often experience difficulties with face recognition. As an example, ASD patients exhibited activation in the right fusiform face area only and no activation of the anterior or posterior cingulate areas during the presentation of familiar faces. Other studies revealed that amygdala activity was bilaterally reduced in individuals with ASD (Tang et al., 2015). Further, altered expression of OXTRs was reported in the right medial temporal lobe in children with ASD (Egawa et al., 2014). It is commonly accepted that children with ASD demonstrate deficits in eye-gaze, facial emotion perception, and face recognition that might be attributed to impaired function of the limbic system. This has also been clearly documented in fMRI studies. Particularly, reduced connectivity between the right fusiform face area and the left amygdala has been found to correspond with the degree of autism severity, and this has also been documented for the connectivity between the right fusiform face area and the right inferior frontal gyrus (Kleinhans et al., 2008). Near-infrared spectroscopy studies have also demonstrated that self-face recognition is also impaired in children with ASD due to dysfunction of the right inferior frontal gyrus area (Kita et al., 2011).

Novel data regarding the mechanisms of face recognition in the human brain have led to new opportunities for the rehabilitation of patients with prosopagnosia (is a cognitive disorder with impaired ability to recognize familiar faces) of various geneses (see these excellent reviews, Bate and Bennetts, 2014; DeGutis et al., 2014). There is evidence of an association between prosopagnosia and the common genetic variants rs53576, rs2254298, and rs237887 in the OXTR gene (Skuse et al., 2014; Cattaneo et al., 2016), although recent findings contradict these reports (Verhallen et al., 2017). Methodology is an important consideration because OT may only affect the temporal element of face recognition, and not accuracy or eye-gaze patterns (Hubble et al., 2017). However, exogenous OT or its stable analogs may, for some individuals with face processing impairment, have the therapeutic potential to enhance face memory (Bate et al., 2014; Blandón-Gitlin et al., 2014; DeGutis et al., 2014). Recent findings indicate that a single administration of OT could increase the allocation of attention toward faces to control levels in patients with ASD. This may be particularly useful for individuals with social anxiety (Kanat et al., 2017).

## Interpersonal Communication and Face Recognition

OT may suppress the ability to inhibit (i.e., ability to ignore task-irrelevant information) the processing of sad faces in individuals with depression (Ellenbogen et al., 2013). In this context, analyses of face perception revealed that empathy predicted poor inter-personal communication in individuals who were unable to inhibit distracting personally relevant facial expressions of anger. Thus, higher levels of empathy could result in a loss of inhibitory control when emotional information is processed (Iacono et al., 2015). This may explain why so-called hypersensitive social cognition is linked with a higher risk of depression development (Harkness et al., 2011). Since it is generally believed that low empathy is associated with low OT levels (Demirci et al., 2016; Deuse et al., 2018), the role of OT in inter-individual communication might be more complex than expected. Empathy is an obligatory aspect of human pro-social behavior that may performed as a collective action (i.e., volunteerism) and is known to be facilitated by OT and affected by OXTR SNPs (Zak and Barraza, 2013).

Face recognition and successful face-to-face interactions are highly important for many types of social communications. A recent non-human primate study reported that a higher frequency of face-to-face interactions between neonates and their mothers positively affected further social behaviors and social interest in the growing infants (Dettmer et al., 2016). Empathy is directly linked to facial perception and recognition, particularly, to the ability to recognize facial expressions. Empathic responses involve activation of the dorsomedial prefrontal area, and this activation is higher in the case of negative stimuli (fearful faces) (Balconi and Bortolotti, 2013). Indeed, empathy might be a trigger for OT release, especially in women (Barraza and Zak, 2009).

## Oxytocin in Clinical Trials

A clinical trial study in which highly functioning individuals with ASD received a single intranasal dose of OT demonstrated that OT facilitated typical and smoother behavioral responses to social communication with conflicting verbal and non-verbal information (Watanabe et al., 2014). fMRI analyses have shown that the behavioral effects of oxytocin are associated with increased brain activity in the anterior cingulate cortex (ACC) and dorsal medial prefrontal cortex (dmPFC), as well as improved functional connectivity from the dmPFC to the ACC (Watanabe et al., 2014; Aoki et al., 2015). These observations are consistent with data from rodent studies showing that oxytocin signaling through OXTR in the medial prefrontal cortex is essential for cognitive flexibility (Insel, 2010; Nakajima et al., 2014; Sabihi et al., 2014, 2017; Albin-Brooks et al., 2017).

Further, a 6-week (OT) intervention in highly functioning individuals with ASD (were given a single dose each week) significantly improved behavioral responses and increased functional connectivity between the ACC and dmPFC (Watanabe et al., 2015). Similarly, administration of a high dose of oxytocin during a 12-week period alleviated symptoms in male young adults with ASD, leading to an increased tendency to visually fixate on regions of social salience such as the eye region of the face, as well as improved biological motion (Kosaka et al., 2016). A study of long-term OT administration in male patients with ASD and comorbid intellectual disability (ID) found that, compared with a placebo, OT significantly increased the frequency of reciprocal social interactions in daily life during the period of administration (Munesue et al., 2016).

Therefore, a number of clinical trials have indicated that OT is able to increase social interaction. This is probably because of increased social identification of faces. However, many of these clinical trials were limited in that they had low numbers of participants and often did not assess functional brain activity during the period of nasal OT administration. OT may have a variable effect depending on the clinical status of the patient (Watanabe et al., 2015; Munesue et al., 2016), oxytocin dosage and genetic background of the OXTR (Kosaka et al., 2016; Munesue et al., 2016), and baseline of plasma OT (Munesue et al., 2016; Yamasue et al., 2018).

Nevertheless clinical trial outcomes together with basic research results provide evidence that OT improves behavioral effects, mutual gaze, face recognition, and mind reading in healthy human individuals as well as ASD patients. So thus accept significant implications for use of OT as a therapy for social impairments in neurodevelopmental disorders.

## Face Recognition Phenomenon in the Era of Advanced Neurotechnologies

Nevertheless active investigations in the area of pharmaceutical therapy of impaired face recognition, especially OT research, other technologies are intensively developed in parallel. Classic transcranial direct current stimulation (tDCS, constant current is applied to the scalp via electrodes), transcranial random noise stimulation (tRNS, weak random current), and galvanic vestibular stimulation (GVS, electric stimulation of the vestibular nerve) alone or in combination with compensatory/remedial training have been shown to improve face recognition performance (Krause and Cohen Kadosh, 2013; Bate and Bennetts, 2014).

The large-scale introduction of neurotechnologies will significantly change the environment in which the human brain develops and functions. In this context, it should be recognized that the mechanisms and consequences of the influence of stimuli in the virtual environment on social behavior, as well as the realization of emotional responses, have not been studied in practice. Recently, interest in this issue has increased dramatically with the development of facial recognition systems and software suitable for the registration of unique biometric parameters of human faces, analysis with existing facial databases, and verification of individuals.

Additional development of neuroimaging techniques and brain–computer interface (BCI)- or optical-imaging-based neurofeedback could further improve the efficacy of rehabilitation approaches to enhancing facial recognition performance (Friedrich et al., 2014; Liu et al., 2016). The latter could be extended to BCI-based technical solutions by using face familiarity within stimulus presentation patterns to elicit face-specific ERP components (i.e., N170) and by utilizing unique brain activity elicited by well-known (familiar) faces. This could increase the speed and fluency of communication for people using a visual stimuli-driven BCI (Zhang et al., 2012; Kashihara, 2014; Yeom et al., 2014; Chen et al., 2015). Combining BCIs with near-infrared spectroscopy systems that have been shown to be sensitive to localized brain dysfunction in individuals with ASD (Iwanaga et al., 2013) could improve signal processing and analysis (Coyle et al., 2007). BCI systems based on the detection of face recognition patterns could be very sensitive to facial emotional processing, and likely also to the empathic component of face processing. Comprehensive investigations regarding the fundamental mechanisms of social and emotional reactions underlying face recognition will facilitate the development and application of virtual reality technologies.

## Conclusion

**FIGURE 3:**
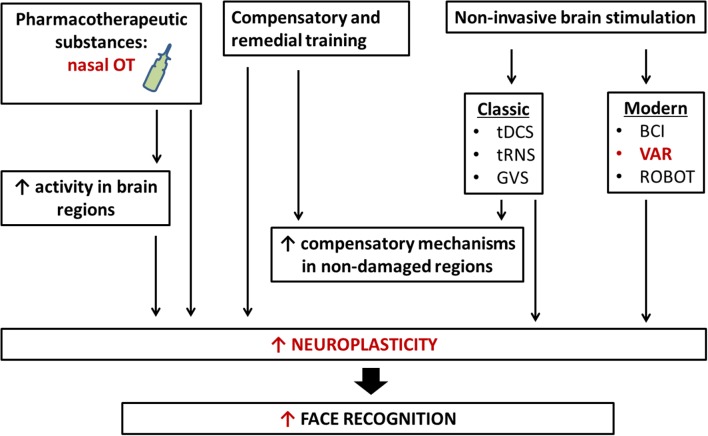
New neurotechnology for rehabilitation of face recognition. Rehabilitation of face recognition includes compensatory and remedial training, the administration of pharmacotherapeutic substances, classic and modern non-invasive brain stimulations, and interaction with robots. The application of more than one technique simultaneously may bring about larger and longer-term benefits. tDCS, transcranial direct current stimulation; tRNS, transcranial random noise stimulation; GVS, galvanic vestibular stimulation; VAR, virtual/adapted reality; BCI, brain–computer interface; OT, oxytocin.

Despite the progress to the stage in understanding the molecular mechanism of face recognition there is no single/simple answer about the best rehabilitation approach to modify face recognition and functionally associated mechanisms in human social behavior. New rehabilitation approaches include different strategies. The first one is an application of various pharmacotherapeutic substances such as OT (via nasal administration). According to the accumulated knowledge, OT has potential and promising future as therapy for abnormal social behavior. So, it is reasonable to speculate that application of OT is a new neurothechnology in treatment of impaired face recognition. On other hand, rapid development of information technologies provides other rehabilitation approaches like transcranial stimulation (or computer-based methods). Direct contact between humans and robots or entities from virtual/additive reality, as well as the wide distribution of social networks based on computer-mediated communication lead to new questions regarding the interchangeability of face-to-face vs. virtual interactions, specifically within the context of human social behavior in real and virtual environments. Progress in humanoid robotics and brain–machine interface (BMI) systems will likely further complicate these issues in the near future. For instance, android robots were recently reported to be useful in assisting and training ASD patients in social interactions (Kumazaki et al., 2017) (**Figure 3**).

Therefore, one may suggest that the OT-mediated effects on face recognition (applied as a supplementary pharmacological support) might be efficiently used within the protocol for BCI systems to enable face-to-face non-verbal communication for people with severe motor and communications impairments, e.g., patients with amyotrophic lateral sclerosis, post-stroke patients, and individuals with ASD. Additionally, there is an intriguing possibility that the autistic brain itself might represent a functional system that is well-adapted for such type of communication, and therefore, some of the ASD-related mechanisms of face perception and processing could be reproduced within BCI systems.

## Author Contributions

OL, YK, YG, HH, and AS conceived the content, and wrote and organized the manuscript. All authors contributed to the manuscript revision, and read and approved the submitted version.

## Conflict of Interest Statement

The authors declare that the research was conducted in the absence of any commercial or financial relationships that could be construed as a potential conflict of interest.
